# Pain in women with and without bipolar spectrum disorder

**DOI:** 10.3389/fgwh.2025.1501382

**Published:** 2025-05-14

**Authors:** Amanda L. Stuart, Michael Berk, Julie A. Pasco, Mohammadreza Mohebbi, Shae E. Quirk, Lana J. Williams

**Affiliations:** ^1^ IMPACT– Institute for Mental and Physical Health and Clinical Translation, School of Medicine, Deakin University, Geelong, VIC, Australia; ^2^ Florey Institute for Neuroscience and Mental Health, University of Melbourne, Parkville, VIC, Australia; ^3^ Orygen, The National Centre of Excellence in Youth Mental Health, Parkville, VIC, Australia; ^4^ School of Public Health and Preventive Medicine, Monash University, Melbourne, VIC, Australia; ^5^ Department of Medicine-Western Health, The University of Melbourne, St Albans, VIC, Australia; ^6^ University Hospital, Geelong, VIC, Australia; ^7^ Biostatistics Unit, Faculty of Health, Deakin University, Burwood, NSW, Australia

**Keywords:** bipolar disorder, pain, comorbidity, mania, depression

## Abstract

**Introduction:**

Bipolar disorder is associated with several physical conditions and possibly increased pain, although research outside hospital settings is limited. We compared perceived pain among population-based women with and without bipolar disorder.

**Method:**

This study examined 113 women with bipolar disorder (59 euthymic, 54 symptomatic in past month) and 316 age-matched women without bipolar disorder drawn from studies located in the same region of south-eastern Australia. Mental disorders were confirmed by clinical interview (SCID-I/NP). Pain during the past week was determined by numeric rating scale (0–10, 10 = pain as severe as I can imagine) and deemed present if ≥5. Demographic, lifestyle, and health information was obtained via questionnaire. Odds ratios (OR) with 95% confidence intervals for the likelihood of pain were estimated using marginal binary logistic regression models, adjusting for potential confounders.

**Results:**

Women with bipolar disorder who were euthymic at the appointment were at increased odds of headache [adjOR 3.4, 95% CI (1.4, 7.9)], back pain [2.6 (1.3, 5.4)], overall pain(s) [5.7 (2.9, 11.4)], pain at ≥3 sites [2.3 (1.0, 5.2)] and were in pain ≥50% time spent awake [2.3 (1.1, 5.1)] compared to women without bipolar disorder. The pattern of association was similar but stronger for women symptomatic in the past month; headache [6.0 (2.6, 13.9)], back pain [4.2 (2.0, 8.5)], overall pain(s) [7.2 (3.4, 15.4)], pain at ≥3 sites [5.1 (2.3, 11.1)] and ≥50% time in pain [4.5 (2.2, 9.3)]. Daily activity interference from pain did not differ between groups (all *p* > 0.05).

**Conclusion:**

Women with bipolar disorder are more likely to report pain regardless of phase. Assessment and management of pain is necessary to reduce associated burden.

## Introduction

Bipolar spectrum disorder, affecting approximately 1.2% of adults, is a chronic mood disorder characterised by fluctuating episodes of mania or hypomania, depression and periods of euthymia (1). Adults with bipolar disorder are at greater risk of mental and medical comorbidities, which can lead to decreased quality of life, higher medical costs, increased mortality and premature death (2–5). There is a growing body of research to suggest the presence of pain is elevated in bipolar disorder (6, 7). Comorbid pain has also been associated with poorer outcomes such as greater sleep disturbances and suicidal behaviour, as well as decreased functioning and quality of life (8, 9).

Stubbs and colleagues conducted a systematic review and meta-analysis investigating the prevalence and nature of pain in bipolar disorder. The review synthesised a total of 22 studies including pooled data from 138,285 individuals with and 12,204,292 without bipolar disorder. The key results indicated that those with bipolar disorder were twice as likely to be at risk of experiencing pain compared to those with no history of bipolar disorder (6). A reported limitation of the evidence included that there is insufficient information on the association between phase of the disorder (e.g., symptomatic vs. euthymic or manic vs. depression) and presence of pain. Furthermore, the majority of included studies utilised samples derived from hospital/medical records and registries.

In the four population-based, observational studies conducted to date, participants with a diagnosis of bipolar disorder were similarly more likely to report chronic pain (10), medically unexplained pain (11), migraines (12), pain at multiple sites (13) and experience at least moderate interference from pain in their everyday activities (14), compared to those without bipolar disorder. However, details regarding whether participants were in a symptomatic or euthymic state when reporting pain were omitted, or not a focus of the study (10–14). Thus, there remains an opportunity to examine the association between different phases of bipolar disorder in relation to pain in a population-based setting. In addition, the burden associated with pain experiences such as time in pain and interference from pain, is yet to be thoroughly investigated in bipolar disorder, whilst also considering symptomatic vs. euthymic phases.

The aim of the current study was to examine excess pain during the euthymic and symptomatic phases of bipolar disorder, relative to those without bipolar disorder, utilising a numerical rating scale to categorise the pain experience, including time in pain and interference from pain. We hypothesise that population-based women with bipolar disorder, particularly those in the symptomatic phase, would be more likely to report pain across all sites that interferes with daily life and is experienced more often compared to those without bipolar disorder.

## Method

### Participants

This study examined data from women residing in the Barwon Statistical Division, a geographically defined region in south-eastern Australia. Women with a diagnosis of bipolar disorder were drawn from the Bipolar Health and Lifestyle study. A detailed description of the study has been published elsewhere (15). Briefly, women aged 20+ years, with bipolar disorder were recruited from healthcare settings (2011–2018) and underwent detailed body composition measures and a battery of health and lifestyle questionnaires. A comparator group, women without bipolar disorder, were drawn from the most recent assessment (2011–2014) for the Geelong Osteoporosis Study (GOS); an ongoing, population-based cohort study, randomly-selected from the electoral rolls (16). The inclusion criteria for GOS participants in the current analyses were: (1) completion of the pain questionnaire (see ‘outcome’ section) and (2) completion of a clinical interview (SCID-I/NP, see “exposures” section) (17) with no history of bipolar disorder or current mood, anxiety or substance use disorder. Participants were age-matched at a ratio of three to one for the 113 women with bipolar disorder, with the exception of the 20–29 year age group for which all available participants (*n* = 19) were included to match the 14 women with bipolar disorder. Thus, 113 women with and 316 women without bipolar disorder were included in this study. All participants provided written informed consent and approval from the Barwon Health Human Research Ethics Committee was obtained (reference numbers 10/89 and 92/01).

### Measures

#### Outcome

Pain experience during the past week was assessed using a Numerical Rating Scale (range 0–10, 0 = no pain and 10 = pain as severe as I can imagine) across four sites: head, back, shoulder and overall pain(s) severity. Amount of time spent in pain during the past week (0–10, 0 = none of the time and 10 = all the time) and the interference with daily activities caused by pain (0–10, 0 = not at all 10 = complete disability) was also captured (18). For these analyses, moderate to severe pain was deemed to be present if scores were ≥5 (19). In addition to the pain questionnaire, participants were asked to indicate the severity of pain at other sites including arm, hand, hip, knee, upper leg, lower leg, ankle, foot, tooth/jaw, sinus, chest, stomach and overall pain(s). Participants with pain at three or more sites were identified.

#### Exposures and covariates

The Structured Clinical Interview for the Diagnostic and Statistical Manual of Mental Disorders, fourth edition (DSM-IV) non-patient edition (SCID-I/NP) (17) was administered to all participants. Lifetime history of bipolar spectrum disorder [I, II and not otherwise specified (NOS)], major depressive disorder, dysthymia, minor depression, mood disorder due to general medical condition, substance induced mood disorder, anxiety disorder (panic disorder, agoraphobia, social phobia, specific phobia, obsessive compulsive disorder, generalised anxiety disorder, anxiety disorder due to a medical condition, substance-induced anxiety disorder and anxiety disorder not otherwise specified) and substance use disorder (any drug or alcohol use disorders) was captured. Using information collected during the SCID-I/NP, participants who experienced a mood, manic or hypomanic episode in the context of bipolar disorder in the past month were further categorised as “symptomatic”; participants who had not experienced a mood, manic or hypomanic episode in the context of bipolar disorder in the past month were categorised “euthymic”. SCID-I/NP interviews were performed by trained staff with qualifications in psychology under the guidance of a psychiatrist.

Height and weight were measured to the nearest 0.1 cm and 0.1 kg, respectively; body mass index (BMI) was calculated (kg/m^2^). Smoking was considered current if participants smoked manufactured or roll your own cigarettes, cigars or a pipe at the time of assessment. Two validated questionnaires were used to determine physical activity; one for participants aged ≥60 years (20) and one for those aged <60 years (21). The questionnaire designed for elderly participants includes items relating to household activities (ten questions), leisure-time (six questions) and sport (two questions) (20). The questionnaire designed for younger adults includes items relating to physical activity at work (eight questions), leisure-time (four questions) and sport (two items) (21). Items relating to leisure-time and sport were calculated using intensity codes based on energetic costs of activities. A domain score was calculated according to the instructions, resulting in three scores (work or home duties, leisure-time and sport). Median scores for our dataset were calculated for each domain in the two questionnaires separately. Participants who scored above the median in each domain were considered active (22). Participants were asked to list medications used at the time of assessment, bringing medication packaging or scripts to assist with recollection. Alcohol (g/day) was estimated via a validated food frequency questionnaire (23). Socioeconomic status (SES) was determined using Socio-Economic Index for Areas (SEIFA) scores based on the Australian Bureau of Statistics Census data at the census closest to and before the study assessment (24). The Index of Relative Socioeconomic Advantage/Disadvantage (IRSAD) was derived from the SEIFA value, accounting for income level and type of occupation and presented in quintiles with quintile 1 indicating the most disadvantaged and quintile 5 the most advantaged.

### Statistical analyses

The distribution of continuous data was visually inspected for parametric test assumptions. Chi square and Kruskal–Wallis tests were used to investigate differences in characteristics across the three groups [bipolar disorder (euthymic), bipolar disorder (symptomatic) and no bipolar disorder] for categorical and non-parametric continuous data, respectively.

To account for the matching structure of the data, a series of binary logistic regression models using Generalised Estimating Equations (GEE) techniques was employed to estimate odds ratios (OR) with 95% confidence intervals (CI) for the likelihood of pain-related outcomes [headache, back pain, shoulder pain, overall pain(s), pain at ≥3 sites, ≥50% of time in pain and ≥50% daily activity interference from pain] among those with bipolar disorder (euthymic) or bipolar disorder (symptomatic) compared to those without bipolar disorder. Both age-adjusted and final models are presented. The selection of confounders included in the final model was guided by the literature (13). The three physical activity measures (work/home duties, leisure-time and sport) were investigated separately to avoid collinearity, with leisure-time chosen for inclusion in the final model due to contributing the most. The final model was adjusted for age, BMI, SES, physical activity during leisure-time, smoking status, alcohol consumption, and use of analgesics and antidepressants, with two-way interactions tested. Statistical analyses were completed using Minitab (version 18) and Stata 17.0 (StataCorp LP. College Station, TX, USA).

## Results

A total of 113 women with a history of bipolar disorder [58 (51.3%) bipolar disorder I, 46 (40.7%) bipolar disorder II and 9 (8.0%) bipolar disorder NOS] and 316 age-matched women with no history of bipolar disorder were included. Of the 113 women with bipolar disorder, 59 were euthymic and 54 were symptomatic [25 (46.3%) mixed episode, 15 (27.7%) manic episode and 13 (24.1%) depressive episode]. One hundred and eighty-one participants reported moderate-severe pain: 102 (32.8%) women without bipolar disorder, 35 (59.3%) with bipolar disorder (euthymic) and 44 (83.0%) with bipolar disorder (symptomatic) (*p* > 0.001).

Table 1 presents participant characteristics. There were no differences between the three groups with regards to age, BMI, SES, physical activity during leisure-time and alcohol consumption, however the women without bipolar disorder were less physically active, less likely to smoke and use both psychotropic and analgesic medications, compared to those with bipolar disorder. Fewer participants without bipolar disorder reported moderate headache, back, shoulder and overall pain, daily activity interference and ≥50% time in pain than the euthymic and the symptomatic bipolar disorder groups.

**Table 1:** Participant characteristics. Data presented as median (interquartile range) or *n* (%).

Variables	All *n* = 429	Bipolar disorder		No bipolar disorder *n* = 316	*P* value
		Euthymic *n* = 59	Symptomatic *n* = 54		
Age, y	49.9 (40.7–57.1)	50.3 (39.2–58.4)	45.3 (39.2–56.2)	50.1 (41.7–57.2)	0.286
BMI, kg/m^2^	27.2 (23.5–32.1)	27.6 (24.6–33.3)	28.1 (24.3–34.5)	27.1 (23.5–31.8)	0.173
Current smoker	74 (17.3)	15 (25.4)	18 (33.3)	41 (13.0)	<0.001
Alcohol consumption, g/day	3.5 (0.4–14.6)	2.6 (0.6–17.9)	3.8 (0.3–12.5)	3.6 (0.4–14.8)	0.975
Socioeconomic status					
Quintile 1 (most disadvantaged)	59 (13.8)	8 (14.4)	8 (14.8)	43 (13.6)	0.715
Quintile 2	54 (12.7)	10 (17.5)	9 (16.7)	35 (11.1)	
Quintile 3	154 (36.1)	21 (36.8)	19 (35.2)	114 (36.1)	
Quintile 4	95 (22.3)	12 (21.1)	13 (24.1)	70 (22.2)	
Quintile 5 (most advantaged)	65 (15.2)	6 (10.5)	5 (9.3)	54 (17.1)	
Physical activity					
Work/home duties	240 (55.9)	39 (66.1)	37 (68.5)	164 (51.9)	0.018
Leisure-time	223 (52.0)	31 (52.5)	21 (38.9)	171 (54.1)	0.117
Sport	247 (57.6)	43 (72.9)	40 (74.1)	164 (51.9)	<0.001
Medication use (current)					
Analgesics	50 (11.7)	10 (17.0)	12 (22.2)	28 (8.9)	0.007
Antidepressants	85 (19.8)	27 (45.8)	29 (53.7)	29 (9.2)	<0.001
Antipsychotics	75 (917.5)	39 (66.1)	35 (64.8)	1 (0.3)	<0.001
Anticonvulsants	48 (11.2)	21 (35.6)	21 (38.9)	6 (1.9)	<0.001
Moderate pain (past week)					
Headache	59 (13.8)	14 (23.7)	21 (38.9)	24 (7.6)	<0.001
Back pain	98 (23.0)	22 (37.3)	28 (51.9)	48 (15.3)	<0.001
Shoulder pain	45 (10.5)	6 (10.2)	12 (22.2)	27 (8.5)	0.010
Overall pain(s)	109 (25.7)	31 (53.5)	33 (63.5)	45 (14.3)	<0.001
Pain at ≥3 sites	75 (17.8)	16 (27.1)	26 (49.1)	33 (10.7)	<0.001
Daily activity interference (≥50%)	49 (11.4)	11 (18.6)	15 (27.8)	23 (7.3)	<0.001
Time in pain (≥50%)	90 (21.0)	19 (32.2)	28 (51.9)	43 (13.6)	<0.001

BMI, body mass index.

Missing data: socioeconomic status *n* = 2, smoking *n* = 1, back pain *n* = 2, overall pain(s) *n* = 5, pain at ≥3 sites *n* = 7.

Table 2 presents the ORs and 95% CIs for age- and fully- adjusted binary logistic regression models.

**Table 2:** Binary logistic regression models investigating the relationship between bipolar disorder and (1) headache, (2) back pain, (3) shoulder pain, (4) overall pain(s), (5) pain at ≥3 sites, (6) daily interference from pain and (7) time in pain.

Variables	Headache		Back pain		Shoulder pain		Overall pain(s)	
	Age-adjusted	Fully adjusted	Age-adjusted	Fully adjusted	Age-adjusted	Fully adjusted	Age-adjusted	Fully adjusted
No bipolar disorder	–	–	–	–	–	–	–	–
Euthymic	3.7 (1.8, 7.7)^#^	3.4 (1.4, 7.9)*	3.3 (1.8, 6.1)^#^	2.6 (1.3, 5.4)*	1.2 (0.4, 3.1)	0.7 (0.2, 2.3)	6.8 (3.7, 12.5)^#^	5.7 (2.9, 11.4)^#^
Symptomatic	7.4 (3.7, 15.0)^#^	6.0 (2.6, 13.9)^#^	6.1 (3.3, 11.3)^#^	4.2 (2.0, 8.5)^#^	3.0 (1.4, 6.5)*	1.4 (0.5, 3.4)	10.1 (5.3, 19.3)^#^	7.2 (3.4, 15.4)^#^
Age	1.0 (1.0, 1.0)	1.0 (0.9, 1.0)	1.0 (1.0, 1.0)	1.0 (1.0, 1.0)	1.0 (0.9, 1.0)	1.0 (0.9, 1.0)	1.0 (0.9, 1.0)	1.0 (0.9, 1.0)
SES		1.1 (0.8, 1.4)		1.3 (1.1, 1.7)*		1.6 (1.2, 2.2)*		1.2 (1.0, 1.5)
Physically active		0.8 (0.5, 1.5)		0.5 (0.3, 0.9)*		0.6 (0.3, 1.1)		0.5 (0.3, 0.8)
Analgesic use		1.3 (0.6, 3.1)		2.4 (1.2, 4.8)*		1.1 (0.4, 3.4)		3.4 (1.6, 7.3)*
Antidepressant use		1.6 (0.7, 3.6)		1.8 (0.9, 3.4)		4.3 (1.8, 10.1)*		1.5 (0.8, 2.9)
BMI		1.0 (1.0, 1.1)		1.0 (1.0, 1.1)		1.0 (0.9, 1.1)		1.0 (1.0, 1.0)
Smoking status		0.7 (0.3, 1.6)		1.4 (0.7,2.5)		1.5 (0.7, 3.2)		1.5 (0.9, 2.6)

	Pain at ≥3 sites		Daily activity interference from pain (≥50%)		Time in pain (≥50%)	
	Age-adjusted	Fully adjusted	Age-adjusted	Fully adjusted	Age-adjusted	Fully adjusted
No bipolar disorder	–	–	–	–	–	–
Euthymic	3.1 (1.6, 6.2)^#^	2.3 (1.0, 5.2)*	2.9 (1.3, 6.4)*	1.5 (0.6, 4.0)	3.0 (1.6, 5.6)^#^	2.3 (1.1, 5.1)*
Symptomatic	8.2 (4.3, 15.6)^#^	5.1 (2.3, 11.1)^#^	4.9 (2.3, 10.3)^#^	2.3 (0.9, 6.1)	6.7 (3.5, 12.5)^#^	4.5 (2.2, 9.3)^#^
Age	1.0 (1.0, 1.0)	1.0 (1.0, 1.0)	1.0 (1.0, 1.0)	1.0 (1.0, 1.0)	1.0 (1.0, 1.0)	1.0 (0.9, 1.0)
SES		1.3 (1.0, 1.7)*		1.0 (0.8, 1.4)		1.4 (1.1, 1.7)*
Physically active		0.8 (0.5, 1.4)		0.7 (0.4, 1.4)		0.9 (0.5, 1.6)
Analgesic use		3.3 (1.6, 6.9)^#^		1.2 (0.5, 2.9)		4.0 (2.0, 8.1)^#^
Antidepressant use		1.9 (0.9, 3.9)		3.2 (1.4, 7.4)*		1.9 (1.0, 3.7)
BMI		1.0 (1.0, 1.1)		1.0 (1.0, 1.1)		1.0 (1.0, 1.1)*
Smoking status		1.6 (0.8, 3.1)		1.4 (0.7, 3.0)		1.1 (0.6, 2.3)

^#^ *p* ≤ 0.001.

* *p* ≤ 0.05.

Generalised estimated equation model to account for the matching nature of the data. SES, socioeconomic status; BMI, body mass index.

Women with bipolar disorder who were euthymic had an approximate three to six - fold increased odds of experiencing headache, back pain, overall pain(s), pain at ≥3 sites, ≥50% daily interference from pain and ≥50% time in pain, compared to those without bipolar disorder (Table 2). Following further adjustment for SES, activity, BMI, smoking and the use of analgesic and antidepressant medications, these relationships were sustained, except for the relationship with daily interference from pain. No relationship was detected between bipolar disorder (euthymia) and shoulder pain.

Women with bipolar disorder who were symptomatic had a three-fold or greater increased odds of experiencing headache, back pain, shoulder pain, overall pain(s), pain at ≥3 sites, daily interference from pain and time in pain, compared to those without bipolar disorder, in age-adjusted models (ORs: 7.4, 6.1, 3.0, 10.1, 8.2, 4.9, 6.7, respectively, Table 2). SES, activity, BMI, smoking and the use of analgesic and antidepressant medications were tested in the models, with the relationships being sustained, with the exception of shoulder pain and daily interference from pain.

## Discussion

In support of our hypotheses, this study demonstrates that women with a diagnosis of bipolar disorder are more likely to report experiencing moderate-severe headache, back pain, overall pain(s), pain at three or more sites and are more likely to report being in pain for at least half of their time awake compared to their age-matched counterparts. These relationships were stronger for women who were symptomatic.

Our findings converge with the existing literature indicating increased pain among those with diagnosed bipolar disorder in clinical and epidemiological studies (6). Akin to studies utilising administrative data (25, 26), we report increased odds of both moderate-severe back pain and headache among those with bipolar disorder. Carney et al. reported 1.5-fold increased odds of back pain and a 2.5- fold increased odds of headache, and Birgenheir et al. reported a 1.9-fold increased odds of back pain and a 2.3-fold increased odds of headache co-occurring with bipolar disorder, compared to control groups (25, 26). Furthermore, euthymic and symptomatic women with bipolar disorder were more likely to report pain at three or more anatomical sites. These findings concur with a large population-based UK study investigating multi-site pain, which found participants who reported multiple sites (both 2–3 and 4–7 sites) of chronic pain were approximately twice as likely to have ‘probable’ bipolar disorder (13). Nicholl and colleagues also reported widespread pain to be more prevalent in those with ‘probable’ bipolar disorder. Several differences between our studies are noted: Nicholl and colleagues investigated chronic pain, classified as pain for a three-month duration, across both men and women aged between 40 and 69 years, utilising self-reported mood symptoms to identify probable mental illness as the outcome in their analyses (13).

In contrast to our hypothesis, we detected no relationship between bipolar disorder status and daily interference from pain. In comparison, Goldstein and colleagues, utilising National Epidemiologic Survey on Alcohol and Related Conditions (NESARC) data, reported participants with a history of bipolar disorder were at 1.6- fold increased likelihood of reporting moderate interference from pain in their everyday activities (14). Differences in study design could explain inconsistencies in results between the two studies. The NESARC study (14) identified participants with an episode of bipolar disorder during the past 12 months using a lay administered questionnaire (Alcohol Use Disorder and Associated Disabilities Interview Schedule) whereas in the current study a lifetime history of bipolar disorder was identified using a structured clinical interview. Moreover, we assessed pain interference over the week prior to assessment, whereas Goldstein et al. enquired about the past four weeks prior to assessment. Finally, we adjusted for activity level, unlike Goldstein et al., and the addition of potential confounders to the model explained the observed association.

Resilience is a likely explanatory factor. When investigating the link between resilience and pain in those with depression, Bauer and colleagues found that a high level of psychological resilience reduced the association between chronic widespread pain and depression, suggesting that interventions designed to increase an individual’s resilience could be useful when dealing with chronic pain (27). It is plausible that participants with higher resilience continue with activities of everyday living with little interruption from pain. Ruiz Parraga and colleagues reported high resilience scores were associated with pain acceptance and improved daily functioning in patients with chronic musculoskeletal back pain (28). Resilience was not measured but could be a key factor in this study.

In contrast to results from a systematic review which reported that a sedentary lifestyle was common among individuals with bipolar disorder (29), participants with bipolar disorder in the current study were found to be more active than those without bipolar disorder with regard to sport and work/household duties. This finding may be indicative of a healthy participant bias; participation in the current study required completion of a comprehensive battery of questionnaires and clinical assessments at the study centre. Furthermore, participants with severe pain may not have been able to attend a study visit, thus results should be interpreted with caution as they may underestimate the likelihood of pain among those with bipolar disorder.

Polarity of episode might be important in pain perception. Boggero and Cole interviewed 201 patients with comorbid chronic pain and bipolar disorder, reporting that 64% of patients recalled reduced pain intensity during recent manic or hypomanic episode (30). However, a smaller qualitative study of 15 veterans with bipolar disorder and chronic pain included accounts of both increased, decreased and disconnected pain experience during manic episodes (31). Interestingly, evidence suggests that those in a current depressive episode are more likely to experience increased pain (32, 33). In the current study we were not powered to analyse by type of episode, but future studies should aim to explore this further.

Bipolar disorder and pain perception are understood to interact through both psychological and biological factors involving the stress response, neurotransmitter modulation and inflammatory pathways. There is considerable overlap between the pathways regulating mood and those in pain perception. The Hypothalamic-Pituitary-Adrenal (HPA) axis is involved in the body’s response to stressors, including painful stimuli through regulating the release of cortisol—the primary stress hormone (34). Abnormalities of the HPA axis activity, like cortisol dysregulation, have been demonstrated in patients with bipolar disorder (34). In addition, neurotransmitter changes play a role in the manifestation of bipolar disorder episodes and pain sensitivity (34, 35). Furthermore, pain sensitivity and bipolar disorder appear to be connected through inflammatory pathways, including a role of pro-inflammatory cytokines in both (36, 37). In terms of psychological mechanisms, pain catastrophizing, or other types of emotional distress may activate the stress response, and perpetuate or exacerbate pain sensitivity and possibly mood symptoms in bipolar disorder (36, 37). Furthermore, medication used in the treatment of bipolar disorder to reduce mood symptoms can exhibit anti-inflammatory properties that could also modulate pain related to neuroinflammation, as well as pain and mood related neurotransmitters such as serotonin and noradrenaline (36, 38). Thus, these overlapping mechanistic pathways between bipolar disorder and pain could explain the observed associations.

Given there is a paucity of data using population-based samples, this is a strength of the current study. A further strength of the study is the use of a semi-structured clinical interview based on the DSM for identifying bipolar disorders, including symptomatic and euthymic phases in the past month; previous studies have used self-report, symptomatology or relied on medical records to identify bipolar disorder. The use of an age-matched sample of women without bipolar disorder drawn from the same geographical region is also a strength, as is the capacity to adjust for multiple lifestyle factors in the analyses. Despite the strengths of our study, it is not without limitations. As participants were recruited from the population and were required to be well enough to attend a study visit and consent to be involved in a research study, the sample may not be representative of all adults with bipolar disorder, especially regarding current illness severity. Furthermore, the DSM-IV rather than the DSM—5 was used to identify bipolar disorder. Also, the use of a different cut-off on the Numerical Rating Scale may provide different results. Further investigation into the cause and duration of pain could be informative. However, we did not collect these data and cannot comment on the acute or chronic nature of the pain. Finally, this study was not powered to investigate pain perception by polarity of mood episode. Future research should focus on clarifying the association between mood episode polarity and pain perception and investigate causality in longitudinal studies, expanding the understanding of the associations reported.

Clinical implications are wide ranging. Treatment of pain can be complex and further complicated by comorbid mental or physical conditions, treatment regimens and associated side effects (39–41). Furthermore, suicide risk has been shown to be increased in the presence of co-occurring chronic pain conditions (8, 9). Managing pain during manic and depressive episodes, needs to be considered. Implementation of strategies such as strict activity scheduling has been considered. Finally, incorporating both mental health and pain management in treatment plans is needed.

In summary, women with bipolar disorder are likely to experience heightened backpain, headache and multisite pain during both euthymic and symptomatic periods. The observed increased likelihood of pain was not accompanied by interference in daily living activities, which could lead to further injury or pain exacerbation. Thus, recognizing pain as a significant comorbidity and in turn implementing comprehensive, multidisciplinary treatment approaches that consider both mental and physical health are imperative.

## Data Availability

The raw data supporting the conclusions of this article will be made available by the authors, without undue reservation.
